# Supplementary material for: Molecular insights into antibody-mediated protection against the prototypic simian immunodeficiency virus
Source: Nat Commun. 2022 Sep 6;13:5236. doi: 10.1038/s41467-022-32783-2 (PMC9446601; doi:10.1038/s41467-022-32783-2)
Supplement: Supplementary file 1 — Supplementary Information File [file 41467_2022_32783_MOESM1_ESM.pdf]

Molecular insights into antibody-mediated protection against the prototypic simian immunodeficiency virus

Fangzhu Zhao, Zachary T. Berndsen, Nuria Pedreño-Lopez, Alison Burns, Joel D. Allen, Shawn Barman, Wen-Hsin Lee, Srirupa Chakraborty, Sandrasegaram Gnanakaran, Leigh M. Sewall, Gabriel Ozorowski, Oliver Limbo, Ge Song, Peter Yong, Sean Callaghan, Jessica Coppola, Kim L. Weisgrau, Jeffrey D. Lifson, Rebecca Nedellec, Thomas B Voigt, Fernanda Laurino, Johan Louw, Brandon C. Rosen, Michael Ricciardi, Max Crispin, Ronald C. Desrosiers, Eva G. Rakasz, David I. Watkins, Raiees Andrabi*, Andrew B. Ward*, Dennis R. Burton*, Devin Sok*

*Corresponding authors: Email: andrabi@scripps.edu; andrew@scripps.edu; burton@scripps.edu; dsok@iavi.org

This PDF file includes:

Supplementary Figures 1-7

Supplementary Table 1-4

a

Rhesus ID	SIVmac239	SIVmacE543 (Mol. Clone)	HIV-2 7312A	SIVcpzPtt	HIV BG505 N332	MLV
r11039	5,385	107,423	131,961	<50	<50	<50
r10027	169	73,260	8,945	<50	<50	<50
r11008	1,562	21,777	52,882	<50	<50	<50
r11002	2,061	35,398	6,653	<50	<50	<50
r04161	351	>150.000	7,348	<50	<50	<50
r10014	213	21,372	4,819	<50	<50	<50
r03145	3,717	38,850	2,486	<50	<50	<50
r10051	3,377	NT	NT	NT	NT	<50
rhBB35	676	NT	NT	NT	NT	<50
r10055	672	NT	NT	NT	NT	<50
r01007	4,101	NT	NT	NT	NT	NT
r09004	325	NT	NT	NT	NT	NT
Rh32888	<30	NT	NT	NT	NT	<30
Rh34118	<30	NT	NT	NT	NT	<30
Rh31186	<30	NT	NT	NT	NT	<30
Rh33519	<30	NT	NT	NT	NT	<30
Rh34620	<30	NT	NT	NT	NT	<30

Neut Titer (1/dilution)
1,000
5,000
20000
50000
100,000

b

c

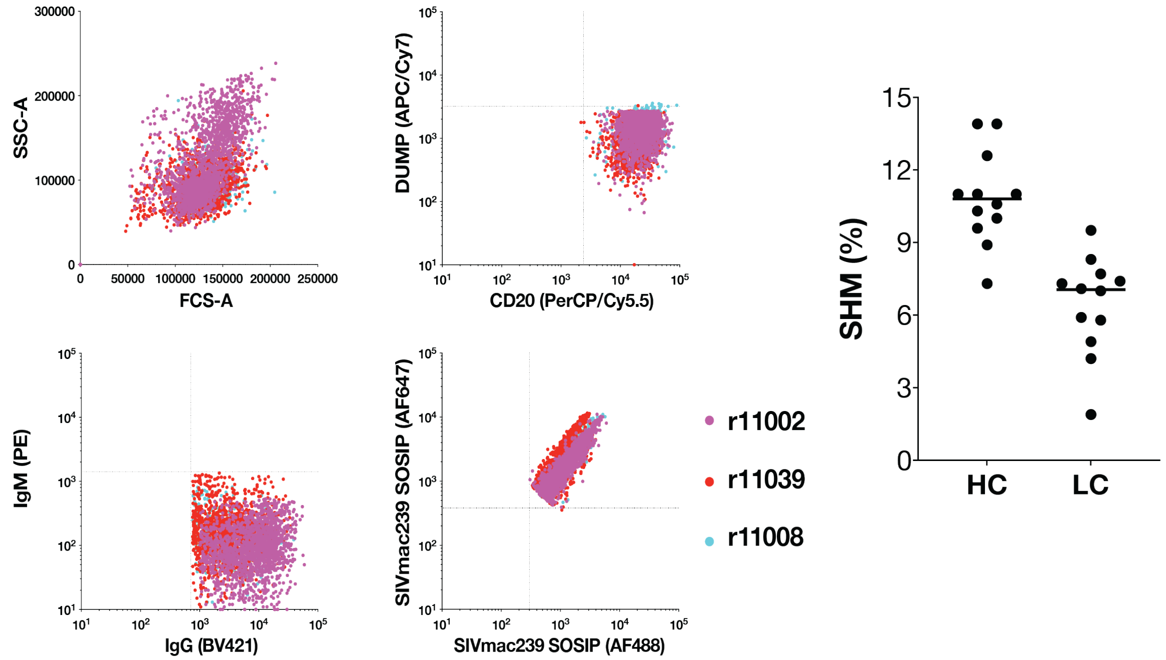

Supplementary Fig.1. Rhesus plasma neutralization titers and FACS sorting of PBMCs from 3 animals. Related to Figure 1. a, Neutralization profile of plasma from SIVmac239 infected rhesus macaques against a panel of pseudoviruses including rhesus macaque SIV (SIVmac239, SIVmacE543), an HIV-2 (HIV-2.7312A), a chimpanzee SIV (SIVcpzPtt), an HIV-1 (BG505), and MLV as a control, showing the ID_{50} of plasma against each virus. N/T: not tested. **b**, Index analysis of single-cell sorted $CD20^{+}IgM^{-}IgG^{+}$SIVmac239 $SOSIP^{++}$ population from the PBMCs of r11002 (pink), r11008 (red), and r11039 (cyan). **c**, Somatic hypermutation (SHM) rates of isolated SIVmac239 mAbs at the nucleotide level.

a

Competitor mAb

mAb ID	J9	FZ019.2	PGT145	CD4-IgaG2	ITS90.03	K11	FZ016.4	FZ020.3	FZ020.7	FZ020.8	5L7	Blank
J9	89	85	-10	7	-16	-15	-6	-14	-14	-9	-1	-9
FZ019.2	77	93	-10	6	-6	-7	-1	-2	-8	-9	-3	-7
PGT145	-4	0	78	9	1	1	2	-1	-2	-4	-2	-5
CD4-IgG2	0	-3	-1	76	-3	-2	-2	-3	-5	-4	-3	-2
ITS90.03	-9	-12	-4	3	83	87	85	75	78	79	3	1
K11	-4	-4	-3	-4	66	83	72	53	52	60	7	-4
FZ016.4	-1	-1	-1	4	77	91	89	76	78	84	2	-1
FZ020.3	-34	-21	-15	14	84	92	89	75	73	80	-11	7
FZ020.7	-1	-2	-4	-2	87	92	92	68	75	79	-1	-4
FZ020.8	1	-11	2	-1	91	91	91	79	80	82	-9	-2
5L7	19	14	6	-15	3	7	-7	-11	-9	-11	84	7

Maximum Competition

- 75-100%
- 50-75%
- 25-50%
- <25%

b

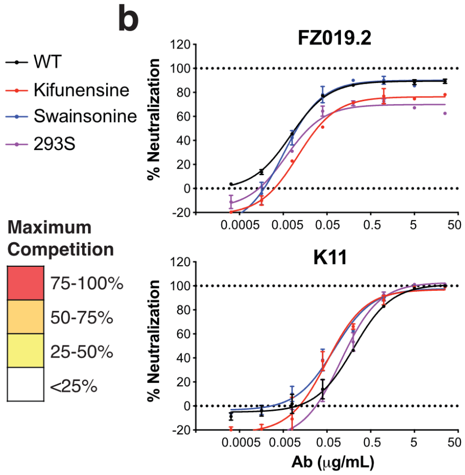

c

Fold Change of IC50	FZ019.2	ITS90.03	K11
**N415A**	6.51	1.30	1.15
**T416A**	KO	1.11	0.88
**N418A**	KO	1.14	1.87
**Q419A**	KO	0.81	0.75
**K420A**	1.59	1.57	1.43
**P421A**	KO	1.18	1.26
**K422A**	1.07	1.13	1.14
**E423A**	2.43	1.03	1.76

d

Competitor mAb

	Blank	5L7	FZ012.7	FZ012.9	FZ012.17	FZ012.18	FZ012.14	FZ012.16	FZ020.10	FZ020.16	K11
5L7	14	81	61	75	81	74	9	2	47	18	10
FZ012.7	-11	81	74	88	87	78	-14	7	24	14	-9
FZ012.9	2	69	57	86	76	67	-11	-15	15	1	-17
FZ012.17	23	72	72	79	80	79	12	-8	15	14	15
FZ012.18	-6	74	61	80	78	76	9	-2	41	19	13
FZ012.14	-6	9	1	4	5	13	78	45	14	3	7
FZ012.16	-9	13	-2	2	-6	8	59	81	4	-6	-14
FZ020.16	8	19	15	11	6	14	7	4	16	87	11
K11	8	1	-4	-8	7	13	2	-17	34	15	82
FZ019.2	-2	4	-8	-14	6	N/A	-17	-10	-6	-8	-15

e

mAb ID	Animal ID	V_H	D_H	J_H	V_H SHM nt (%)	CDRH3	V_L	J_L	V_H SHM nt (%)	CDRL3
FZ012.7	R11039	IGHV4-AFB*01	IGHD2-13*01	IGHJ2-I1	5.74	ARVTLSSWYFDL	LJI.Rh_IGLV3.32	IGLJ1-S1	4.86	QVWDGNTDHYI
FZ012.9	R11039	IGHV4-AFB*01	IGHD2-2*01	IGHJ4*01	6.71	ARVVLAAPYFDS	IGLV3-AED*06	IGLJ2-S2	8.48	QVWDGDSDQYI
FZ012.14	R11039	IGHV1-AAU*01_S5608	IGHD2-35*01	LJI.Rh-IGHJ5.4	7.53	ATLGGSCTGGVCRDYFEF	IGLV7-AAK*01	IGLJ2-S2	12.37	LVYYSGSGL
FZ012.16	R11008	IGHV3-ADL*01	IGHD2-35*01	IGHJ4*01	10.74	ARAGGCIAGVCSYFDS	LJI.Rh_IGKV2.71.a	IGKJ5-I5	5.44	GQGTHWPWT
FZ012.17	R11008	LJI.Rh_IGHV3.141	IGHD1-1*02	LJI.Rh-IGHJ5.4	13.54	TSLSLAGMDDSAEYFQF	IGLV1-ACN*02	IGLJ3*01	4.86	QSYDRTLDIHI
FZ012.18	R11008	LJI.Rh_IGHV3.141	IGHD2-2*01	IGHJ3*01	13.54	TTLSLAGMDDDAEYFVF	IGLV1-S30	IGLJ3*01	4.15	QSYDNGLNIHL
FZ020.16	R11008	IGHV4-ABB*01_S8200	IGHD2-I3	IGHJ5-1*01	12.67	AKEYCKIPSCSSIPV	IGLV3-AEB*02	IGLJ2-S2	6.60	YSTDSSGNHGL

f

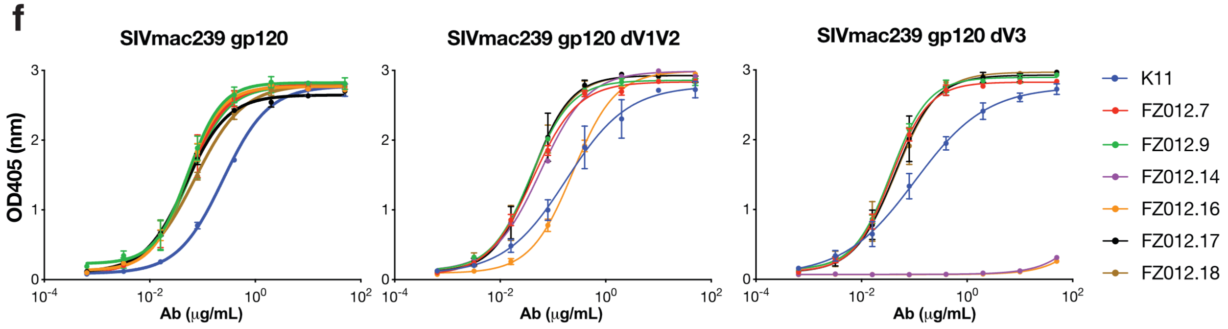

Supplementary Fig.2. Characterization of SIVmac239 nAb and non-nAb epitope specificities. Related to Figure 2 and Figure 3. a, ELISA competition between SIVmac239 nAbs, a SIVmac239 non-nAb 5L7, an HIV-1 bnAb PGT145, and CD4-IgG2 for binding to SIVmac239 SOSIP.664. Maximum percentage of competition was colored

according to the key. **b**, Representative neutralization curves of FZ019.2 and K11 against wildtype SIVmac239 virus, 293S-generated virus, and glycosidase inhibitor-treated viruses at the following concentrations: 25 μM kifunensine and 20 μM swainsonine, respectively. Data are presented as mean values +/- SD, and error bars are from two technical replicates. **c**, Fold reduction of neutralization potency against alanine-scan variants at the V4 loop relative to wild type SIVmac239 pseudovirus. KO: knockout of neutralization i.e. no neutralization measured at 50 μg/mL of mAbs. **d**, ELISA competition between SIVmac239 non-nAbs, 5L7, as well as nAbs K11 and FZ019.2 for binding to SIVmac239 SOSIP.664. Maximum percentage of competition was colored according to the key. **e**, Immunogenetics of SIVmac239 non-nAb mAbs. Rhesus mAbs were annotated with a new rhesus germline database[60]. **f**, Representative ELISA binding curves of non-nAbs binding to SIVmac239 gp120, gp120 dV1V2, and gp120 dV1V2V3. Antibodies were colored according to the key. Data are presented as mean values +/- SD, and error bars are from two technical replicates. Data are representative for at least two independent experiments.

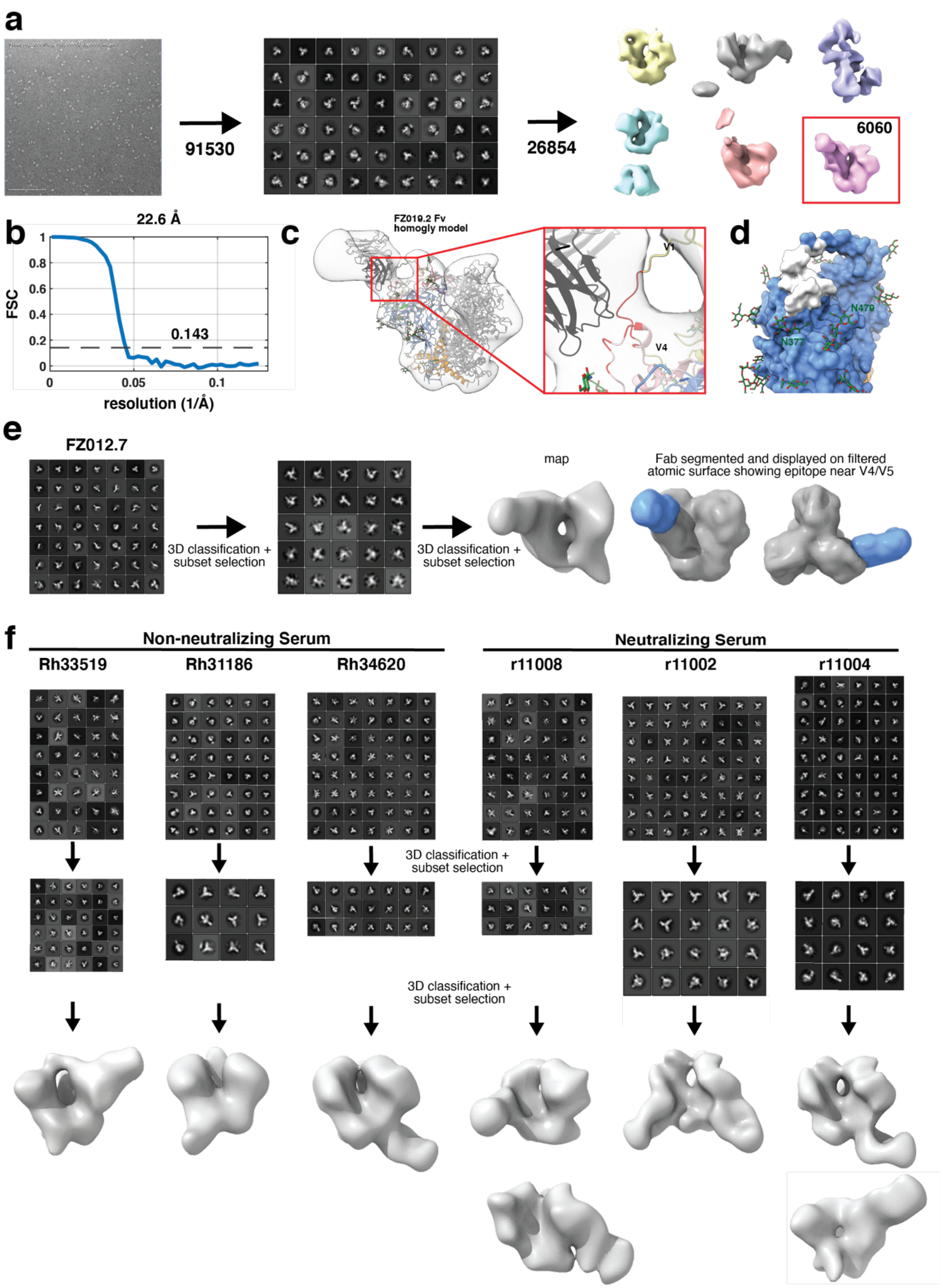

a
91530
26854
6060
b
22.6 Å
FSC
0.143
resolution (1/Å)
c
FZ019.2 Fv
homogly model
V1
V4
d
N479
N377
e
FZ012.7
3D classification +
subset selection
3D classification +
subset selection
map
Fab segmented and displayed on filtered
atomic surface showing epitope near V4/V5
f
Non-neutralizing Serum
Neutralizing Serum
Rh33519
Rh31186
Rh34620
r11008
r11002
r11004
3D classification +
subset selection
3D classification +
subset selection

Supplementary Fig.3. NSEM data processing workflows all mAb and polyclonal data. Related to Figure 1 and Figure 2. a, Data processing workflow for FZ019.2 Fab bound to SIVmac239 SOSIP showing a representative raw micrograph along with 2-D and 3-D class averages and particle numbers indicated at each step. **b**, Fourier Shell Correlation plot for the final 3-D reconstruction. **c**, FZ019.2 epitope mapping using the cryo-EM structure and homology model of the Fab showing potential epitope residues located in the V1 and V4 loops colored in red. **d**, Molecular surface representation of SIVmac239 gp120 showing FZ019.2 epitope in white. **e**, Data processing workflow for FZ012.7 bound to the SIVmac239 trimer along with a segmentation of the Fab displayed on a molecular surface representation of the SIVmac239 trimer structure. **f**, Data processing workflow for all polyclonal samples tested.

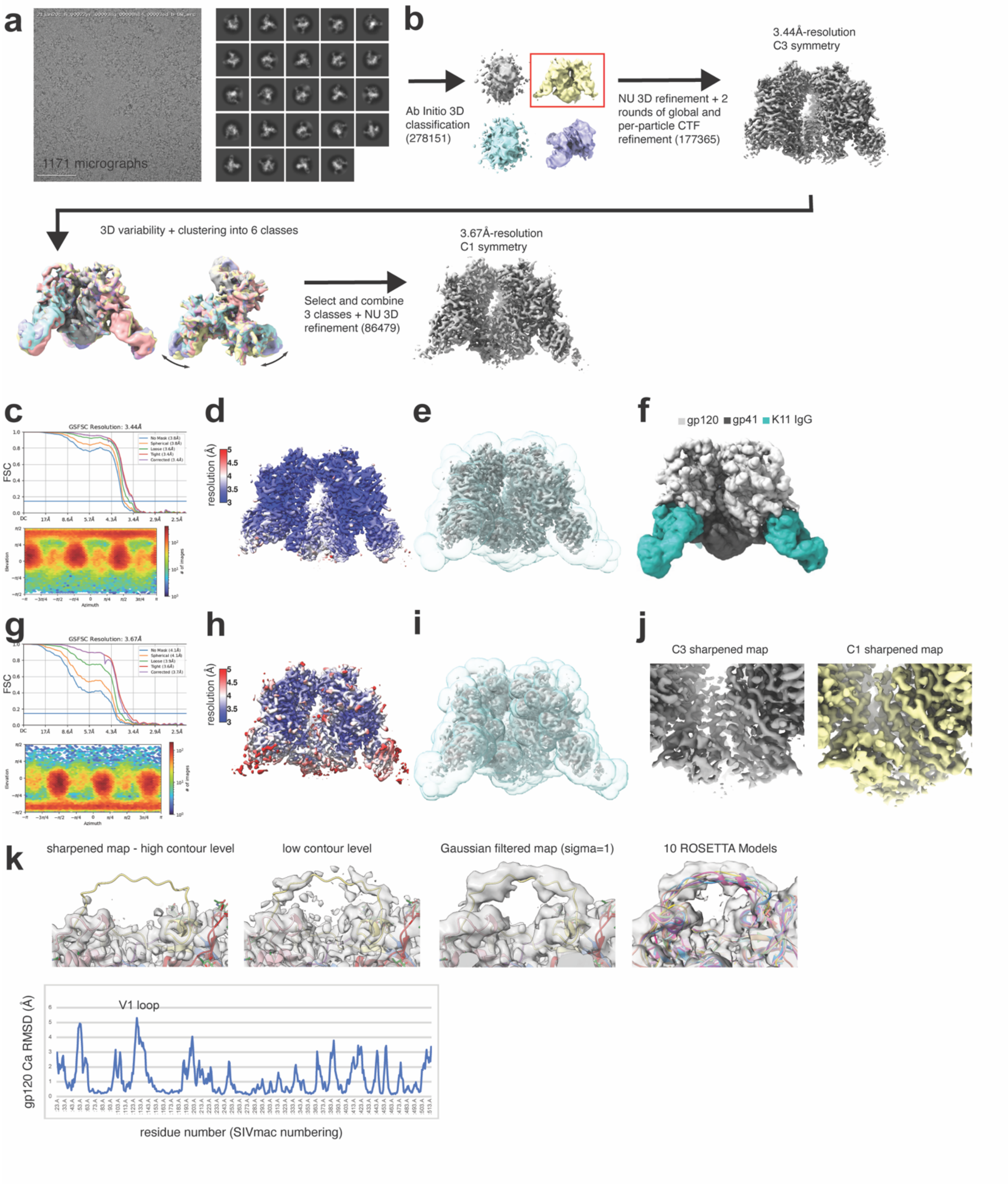

Supplementary Fig.4. Cryo-EM data processing workflow. Related to Figure 4. a, Raw micrograph and representative 2-D class averages for the SIVmac239.K180S + K11 IgG cryo-EM dataset. **b**, 3-D data processing workflow showing sorting by *ab initio* classification and 3-D variability analysis resulting in a C3 consensus refinement and

asymmetric refinement from 3D variability clusters. **c**, Fourier Shell Correlation and angular distribution plots for the C3 consensus refinement. **d**, Local resolution analysis, **e**, mask used for refinement, and **f**, final segmented map. **g-i**, Same as **c-d** but for asymmetric refinement. **j**, Close up view of map density corresponding to gp41 in the C3 symmetric and asymmetric maps showing more ordering in the asymmetric pose. **k**, Close up views of the extended V1 loop segment between residues ~127-139 showing map density in the 3.4Å sharpened map and a Gaussian filtered map (sigma=1) at high and low contour levels along the refined atomic model and the top 10 scoring models from ROSETTA fragment-based refinement. Shown below is the alpha carbon RMSD for 100 ROSETTA models with the extended V1 loop labeled.

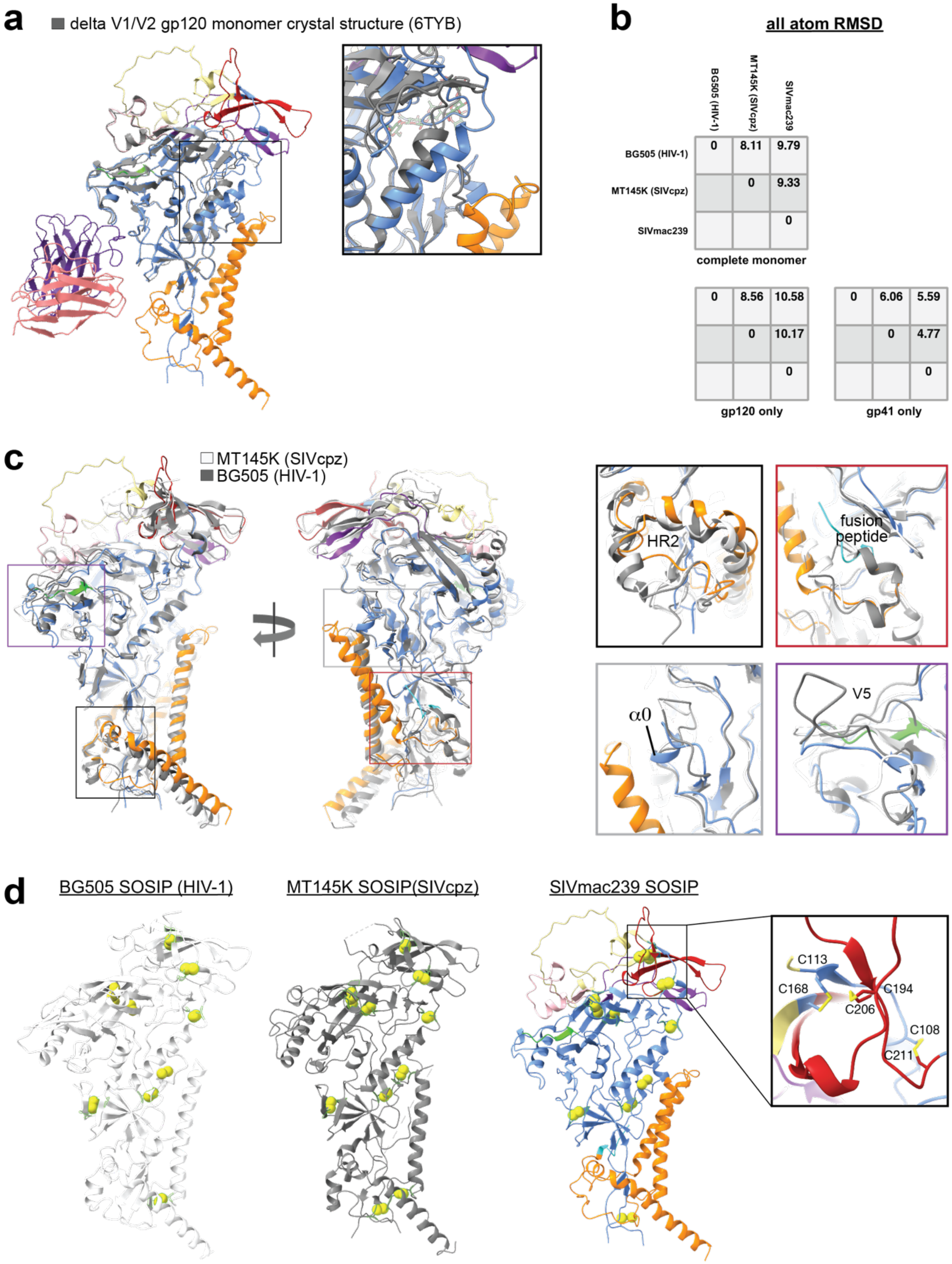
a
delta V1/V2 gp120 monomer crystal structure (6TYB)
b
all atom RMSD
BG505 (HIV-1)
MT145K (SIVcpz)
SIVmac239
BG505 (HIV-1)
MT145K (SIVcpz)
SIVmac239
0
8.11
9.79
0
9.33
0
complete monomer
0
8.56
10.58
0
10.17
0
gp120 only
0
6.06
5.59
0
4.77
0
gp41 only
c
MT145K (SIVcpz)
BG505 (HIV-1)
HR2
fusion peptide
α0
V5
d
BG505 SOSIP (HIV-1)
MT145K SOSIP(SIVcpz)
SIVmac239 SOSIP
C113
C168
C194
C206
C108
C211

Supplementary Fig.5. Extended structural analysis. Related to Figure 4. a, Alignment of our cryo-EM structure with the delta V1/V2 gp120 monomer crystal structure (PDB: 6TYB) showing close agreement outside of the trimerization and gp120-gp41 interfaces (close-up view). **b**, All atom root mean squared deviations (RMSD) between all three structures. **c**, Two views of the SIVmac239 monomer with MT145K SIVcpz and BG505 SOSIP.664 HIV-1 Env structures overlayed and aligned to gp120. Coloring the same as Figure 4. Close up views from designated regions in panel A with higher levels of structural variability between the three models. Specifically, the HR2 helical turn, fusion peptide, alpha 0, and V5 loop. **d**, Detected (highlighted in green) and probable (not highlighted) disulfide bonds for all three models. Close up of the base of V1 and V2 loops showing the additional disulfide unique to SIVmac239.

a

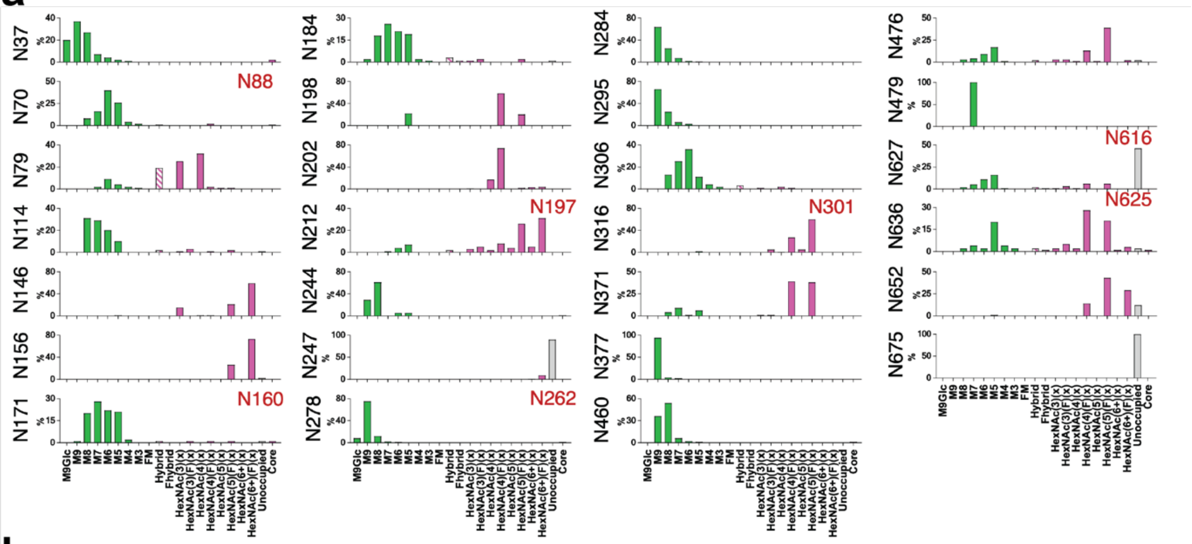

b

	N37	N70	N79	N114	N146	N156	N171	N184	N198	N202	N212	N244	N247	N278	N284	N295	N306	N316	N371	N377	N460	N476	N479	N627	N636	N652	N675
Oligomannose	97	95	18	90	2	0	95	89	22	0	13	99	0	99	100	100	91	2	21	100	98	34	100	35	33	1	0
Hybrid	0	1	20	2	0	0	1	4	0	0	2	0	0	0	0	0	3	0	0	0	0	2	0	2	3	0	0
Complex	3	4	62	6	98	98	4	5	78	100	85	1	10	1	0	0	6	98	79	0	2	62	0	16	62	87	0
Unoccupied	0	0	0	1	0	2	1	1	0	0	0	0	90	0	0	0	0	0	0	0	0	2	0	46	2	12	100
Fucose	0	3	4	5	81	98	2	5	78	79	70	0	9	0	0	0	2	93	79	0	0	58	0	15	58	87	0
NeuAc	0	0	7	0	47	2	0	0	0	25	14	0	0	0	0	0	1	23	13	0	0	11	0	9	10	21	0
Sulfated	0	0	0	0	0	0	0	0	0	0	4	0	10	0	0	0	0	0	0	0	0	0	0	0	1	3	0

c

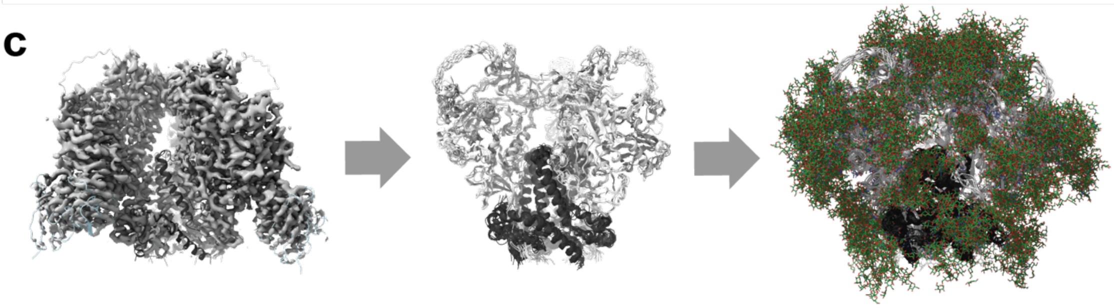

1. Build complete atomic model into cryo-EM map wihtout glycans

2. Generate a diverse set of protein models with Rosetta multicycle refinement and select the top ten scoring models to serve as scaffolds (removing Fab chains if present)

3. Build and relax 100 fully glycosylated models for each protein scaffold with ALLOSMOD using the most common glycoform at each site as determined by MS (1000 models total).

d

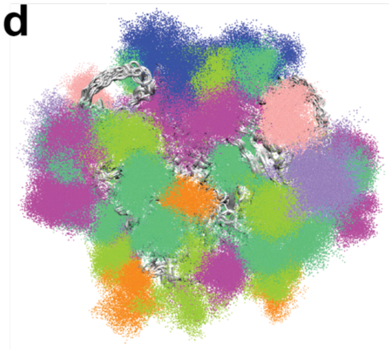

Supplementary Fig.6. Extended glycan shield analysis. Related to Figure 5. a, Complete site-specific MS analysis showing the distribution of specific glycoforms for each PNGS along with gross percentages of each type of glycan **b**. **c**, Schematic overview of the modified HTAM pipeline using the cryo-EM map to guide the generation of protein scaffolds for subsequent glycan modeling**. d**, All fully glycosylated models aligned to one another with glycans colored according to their type designated in the legend.

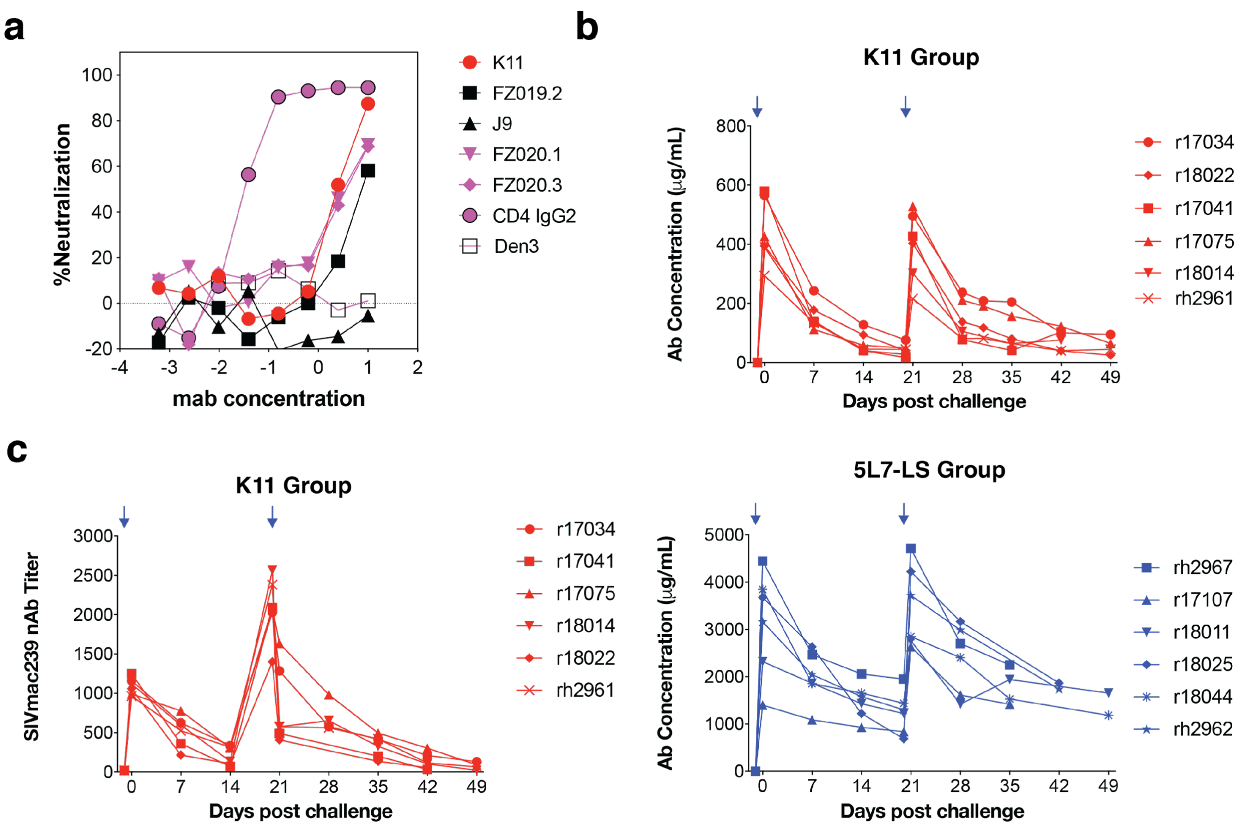

Supplementary Fig.7. Macaque challenge study details. Related to Figure 6. a, Neutralization curves of K11 and FZ019.2 against SIVmac239 challenge virus. CD4-IgG2 was the positive control and Den3 was negative control. Antibodies are colored according to the key. **b**, Plasma antibody concentrations in the K11 group (top) and 5L7-LS group (bottom). Rhesus plasma 5L7 was measured by ELISA binding to the C9 tag on the 5L7-LS antibody whereas K11 was measured by ELISA binding to SIVmac239 gp140 FT. Arrow represents antibody infusion. **c**, Plasma SIVmac239 nAb titers in the K11 group. Data are representative for two independent experiments.

Supplementary Table 1. Mapping SIVmac239 mAb binding specificities by ELISA.

mAb ID	Animal ID	SIVmac239 ELISA Binding EC_{50} (μg/mL)					
		SOSIP	gp140 FT	FT dV1V2V3	gp120	gp120 dV1V2	gp120 dV3
K11	r11008	0.529	0.100	0.066	0.109	0.055	0.068
FZ012.15	r11008	0.611	0.077	0.037	0.058	0.036	0.062
FZ020.1	r11008	0.337	0.063	0.044	0.045	0.034	0.053
FZ020.3	r11008	0.226	0.031	0.042	0.034	0.036	0.053
FZ020.5	r11008	1.799	0.092	0.023	0.099	0.067	0.127
FZ016.3	r11002	1.130	0.050	0.028	0.070	0.088	0.245
FZ016.4	r11002	0.411	0.033	0.022	0.039	0.040	0.089
FZ016.5	r11002	0.669	0.047	0.027	0.048	0.070	0.176
FZ020.7	r11002	0.923	0.042	0.022	0.067	0.079	0.113
FZ020.8	r11002	0.958	0.040	0.021	0.064	0.064	0.109
FZ019.2	r11039	0.334	0.527	>50	2.752	>50	1.159
J9	r11039	0.084	1.158	>50	N/A	>50	N/A
**ITS90.03**	N/A	0.289	0.047	0.043	0.028	0.022	0.040

Supplementary Table 2. SIVmac239 Env 15-mer overlapping peptide sequences.

Epitope	Biotinylated Peptide ID	Sequence	Epitope	Biotinylated Peptide ID	Sequence
V1V2	SIVmac239_Env_Peptide1	NKSETDRWGLTKSIT	gp41	SIVmac239_Env_Peptide39	GVFVLGFLGFLATAG
V1V2	SIVmac239_Env_Peptide2	DRWGLTKSITTTAST	gp41	SIVmac239_Env_Peptide40	GFLGFLATAGSAMGA
V1V2	SIVmac239_Env_Peptide3	TKSITTTASTTSTTA	gp41	SIVmac239_Env_Peptide41	LATAGSAMGAASLTL
V1V2	SIVmac239_Env_Peptide4	TTASTTSTTASAKVD	gp41	SIVmac239_Env_Peptide42	SAMGAASLTLTAQSR
V1V2	SIVmac239_Env_Peptide5	TSTTASAKVDMVNET	gp41	SIVmac239_Env_Peptide43	ASLTLTAQSRTLLAG
V1V2	SIVmac239_Env_Peptide6	SAKVDMVNETSSCIA	gp41	SIVmac239_Env_Peptide44	TAQSRTLLAGIVQQQ
V1V2	SIVmac239_Env_Peptide7	MVNETSSCIAQDNCT	gp41	SIVmac239_Env_Peptide45	TLLAGIVQQQQQLLD
V1V2	SIVmac239_Env_Peptide8	SSCIAQDNCTGLEQE	gp41	SIVmac239_Env_Peptide46	IVQQQQQLLDVVKRQ
V1V2	SIVmac239_Env_Peptide9	QDNCTGLEQEQMISC	gp41	SIVmac239_Env_Peptide47	QQLLDVVKRQQELLR
V1V2	SIVmac239_Env_Peptide10	GLEQEQMISCKFNMT	gp41	SIVmac239_Env_Peptide48	VVKRQQELLRLTVWG
V1V2	SIVmac239_Env_Peptide11	QMISCKFNMTGLKRD	gp41	SIVmac239_Env_Peptide49	QELLRLTVWGTKNLQ
V1V2	SIVmac239_Env_Peptide12	KFNMTGLKRDKKKEY	gp41	SIVmac239_Env_Peptide50	LTVWGTKNLQTRVTA
V1V2	SIVmac239_Env_Peptide13	GLKRDKKKEYNETWY	gp41	SIVmac239_Env_Peptide51	TKNLQTRVTAIEKYL
V1V2	SIVmac239_Env_Peptide14	KKKEYNETWYSADLV	gp41	SIVmac239_Env_Peptide52	TRVTAIEKYLKDQAQ
V1V2	SIVmac239_Env_Peptide15	NETWYSADLVCEQGN	gp41	SIVmac239_Env_Peptide53	IEKYLKDQAQLNAWG
V1V2	SIVmac239_Env_Peptide16	SADLVCEQGNNTGNE	gp41	SIVmac239_Env_Peptide54	KDQAQLNAWGCAFRQ
V1V2	SIVmac239_Env_Peptide17	CEQGNNTGNESRCYM	gp41	SIVmac239_Env_Peptide55	LNAWGCAFRQVCHTT
V1V2	SIVmac239_Env_Peptide18	NTGNESRCYMNHCNT	gp41	SIVmac239_Env_Peptide56	CAFRQVCHTTVPWPN
V3	SIVmac239_Env_Peptide19	RRPGNKTVLPVTIMS	gp41	SIVmac239_Env_Peptide57	VCHTTVPWPNASLTP
V3	SIVmac239_Env_Peptide20	KTVLPVTIMSGLVFH	gp41	SIVmac239_Env_Peptide58	VPWPNASLTPKWNNE
V3	SIVmac239_Env_Peptide21	VTIMSGLVFHSQPIN	gp41	SIVmac239_Env_Peptide59	ASLTPKWNNETWQEW
V3	SIVmac239_Env_Peptide22	GLVFHSQPINDRPKQ	gp41	SIVmac239_Env_Peptide60	KWNNETWQEWERKVD
V3	SIVmac239_Env_Peptide23	SQPINDRPKQAWCWF	gp41	SIVmac239_Env_Peptide61	TWQEWERKVDFLEEN
V4	SIVmac239_Env_Peptide24	KMNWFLNWVEDRNTA	gp41	SIVmac239_Env_Peptide62	ERKVDFLEENITALL
V4	SIVmac239_Env_Peptide25	LNWVEDRNTANQKPK	gp41	SIVmac239_Env_Peptide63	FLEENITALLEEAQI
V4	SIVmac239_Env_Peptide26	DRNTANQKPKEQHKR	gp41	SIVmac239_Env_Peptide64	ITALLEEAQIQQEKN
V4	SIVmac239_Env_Peptide27	NQKPKEQHKRNYVPC	gp41	SIVmac239_Env_Peptide65	EEAQIQQEKNMYELQ
V5	SIVmac239_Env_Peptide28	WIDGNQ	gp41	SIVmac239_Env_Peptide66	QQEKNMYELQKLNSW
C3	SIVmac239_Env_Peptide29	CWFGGKWKDAIKEVK	gp41	SIVmac239_Env_Peptide67	MYELQKLNSWDVFGN
C3	SIVmac239_Env_Peptide30	KWKDAIKEVKQTIVK	gp41	SIVmac239_Env_Peptide68	KLNSWDVFGNWFDLA
C3	SIVmac239_Env_Peptide31	IKEVKQTIVKHPRYT	gp41	SIVmac239_Env_Peptide69	DVFGNWFDLASWI
C3	SIVmac239_Env_Peptide32	QTIVKHPRYTGTNNT			
C3	SIVmac239_Env_Peptide33	HPRYTGTNNTDKINL			
C3	SIVmac239_Env_Peptide34	GTNNTDKINLTAPGG			
C3	SIVmac239_Env_Peptide35	DKINLTAPGGGDPEV			
C3	SIVmac239_Env_Peptide36	TAPGGGDPEVTFMWT			
C3	SIVmac239_Env_Peptide37	GDPEVTFMWTNCRGE			
C3	SIVmac239_Env_Peptide38	TFMWTNCRGEFLYCK			

Supplementary Table 3. Cryo-EM data and atomic model statistics.

	**SIVmac239.K180S SOSIP + K11 IgG, C3 (Cryo-EM)**	**SIVmac239.K180S SOSIP + K11 IgG, C1 (Cryo-EM)**
	**EMDB: EMD-25621)**	**EMDB: EMD-25676**
	**PDB ID: 7T2P**	**PDB ID: 7T4G**
**Data Collection and Processing**		
Electron microscope	Titon Krios	Titon Krios
Electron detector	K2 Summit	K2 Summit
Magnification	36,000	36,000
Voltage (kV)	200	200
Electron exposure (e–/Å^2)	5	5
Defocus range (μm)		
Pixel Size (Å)	1.15	1.15
Symmetry imposed	C3	C1
Initial particle images (no.)	278,151	278,151
Final particle images (no.)	177,365	86,479
Map resolution (Å)	3.47	3.67
FSC threshold	0.143	0.143
Map sharpening *B* factor (Å^2)	101.8	127.3
**Model building and refinement**		
Initial models used	(6TYB,6X9V)	C1 Map
Model composition		
Protein Chains	4	12
Protein Residues	873	2619
Ligands	56	176
R.m.s. deviations		
Bond Lengths (Å)	0.021	0.021
Bond angles (°)	1.816	1.771
Ramachandran plot		
Favored (%)	95.49	95.8
Disallowed (%)	1.39	1.35
Validation		
MolProbity score	1.09	1.12
Clashscore	0.93	1.17
Poor rotamers (%)	0.39	0.26
EMRinger score	2.06	1.87
Map-model cross correlation	0.79	0.82
CaBLAM outliers (%)	0.12	3.66

Supplementary Table 4. Genetic background of animals in the challenge study.

Group	ID	Sex	MHC	Age (years)
Group 1 5L7-LS	rh2967	female	A*02-	4.88
Group 1 5L7-LS	r17107	male	A*02+	3.30
Group 1 5L7-LS	r18011	female	A*02+	2.96
Group 1 5L7-LS	r18025	male	A*02-	2.83
Group 1 5L7-LS	r18044	male	A*02+	2.72
Group 1 5L7-LS	rh2962	female	A*02-	3.67
Group 2 K11	r17034	male	A*02-	3.75
Group 2 K11	r17041	female	A*02-	3.71
Group 2 K11	r17075	female	A*02-	3.45
Group 2 K11	r18014	male	A*02-	2.93
Group 2 K11	r18022	male	A*02+	2.88
Group 2 K11	rh2961	female	A*02-	4.82
Group 3	r17068	male	A*02-	3.55
Group 3	r17024	male	A*02-	3.79
Group 3	r17035	male	A*02-	3.75
Group 3	r17060	male	A*02+	3.59
Group 3	r18017	male	A*02+	2.92
Group 3	rh2751	female	A*02-	5.78
