# Supplementary material for: Molecular insights into antibody-mediated protection against the prototypic simian immunodeficiency virus
Source: Nat Commun. 2022 Sep 6;13:5236. doi: 10.1038/s41467-022-32783-2 (PMC9446601; doi:10.1038/s41467-022-32783-2)
Supplement: Supplementary file 3 — Description of Additional Supplementary Information [file 41467_2022_32783_MOESM3_ESM.pdf]

Description of Additional Supplementary Information

Title: Supplemental Movie 1.

Description: CryoSparc 3-D variability analysis of SIVmac239 SOSIP trimer. Map morphs showing heterogeneity associated with the first two eigenvectors viewed from the side and top of the SIVmac239 SOSIP trimer.
