# Supplementary material for: Molecular insights into antibody-mediated protection against the prototypic simian immunodeficiency virus
Source: Nat Commun. 2022 Sep 6;13:5236. doi: 10.1038/s41467-022-32783-2 (PMC9446601; doi:10.1038/s41467-022-32783-2)
Supplement: Supplementary file 5 — Reporting Summary [file 41467_2022_32783_MOESM5_ESM.pdf]

nature portfolio

Corresponding author(s): Raiees Andrabi, Andrew B. Ward, Dennis R. Burton, Devin Sok

Last updated by author(s): Jul 1, 2022

Reporting Summary

Nature Portfolio wishes to improve the reproducibility of the work that we publish. This form provides structure for consistency and transparency in reporting. For further information on Nature Portfolio policies, see our Editorial Policies and the Editorial Policy Checklist.

Statistics

For all statistical analyses, confirm that the following items are present in the figure legend, table legend, main text, or Methods section.

n/a	Confirmed	
☐	☒	The exact sample size (n) for each experimental group/condition, given as a discrete number and unit of measurement
☐	☒	A statement on whether measurements were taken from distinct samples or whether the same sample was measured repeatedly
☐	☒	The statistical test(s) used AND whether they are one- or two-sided<br>*Only common tests should be described solely by name; describe more complex techniques in the Methods section.*
☒	☐	A description of all covariates tested
☒	☐	A description of any assumptions or corrections, such as tests of normality and adjustment for multiple comparisons
☐	☒	A full description of the statistical parameters including central tendency (e.g. means) or other basic estimates (e.g. regression coefficient) AND variation (e.g. standard deviation) or associated estimates of uncertainty (e.g. confidence intervals)
☐	☒	For null hypothesis testing, the test statistic (e.g. F, t, r) with confidence intervals, effect sizes, degrees of freedom and P value noted<br>*Give P values as exact values whenever suitable.*
☒	☐	For Bayesian analysis, information on the choice of priors and Markov chain Monte Carlo settings
☒	☐	For hierarchical and complex designs, identification of the appropriate level for tests and full reporting of outcomes
☒	☐	Estimates of effect sizes (e.g. Cohen's d, Pearson's r), indicating how they were calculated

Our web collection on statistics for biologists contains articles on many of the points above.

Software and code

Policy information about availability of computer code

Data collection	Negative stain EM and Cryo-EM data collection was performed using the Leginon software package; All movie micrographs were aligned and dose-weighted using MotionCor2 and CTF parameters were estimated with GCTF. Single-particle processing was carried out using CryoSparc2.
Data analysis	All single-particle data processing steps were performed with either CryoSparc2 (CryoEM) or Relion/3.0 (nsEM). Model building was performed using SWISS-MODEL, SAbPred, COOT, and Rosetta.

For manuscripts utilizing custom algorithms or software that are central to the research but not yet described in published literature, software must be made available to editors and reviewers. We strongly encourage code deposition in a community repository (e.g. GitHub). See the Nature Portfolio guidelines for submitting code & software for further information.

Data

Policy information about availability of data

All manuscripts must include a data availability statement. This statement should provide the following information, where applicable:

- Accession codes, unique identifiers, or web links for publicly available datasets
- A description of any restrictions on data availability
- For clinical datasets or third party data, please ensure that the statement adheres to our policy

Antibody sequences are available on Genbank (ON526845-ON526868). Cryo-EM and nsEM structures and refined atomic models have been deposited to the Electron Microscopy Data Bank under accession codes EMD-25621, EMD-25676, EMD-25623, EMD-25624, EMD-25625, EMD-25626, EMD-25627, EMD-25628, EMD-25629, EMD-25630, EMD-25631, and EMD-25632, and the Protein Data Bank under accession codes 7T2P and 7T4G.

March 2021

Field-specific reporting

Please select the one below that is the best fit for your research. If you are not sure, read the appropriate sections before making your selection.

- [x] Life sciences
- [ ] Behavioural & social sciences
- [ ] Ecological, evolutionary & environmental sciences

For a reference copy of the document with all sections, see nature.com/documents/nr-reporting-summary-flat.pdf

Life sciences study design

All studies must disclose on these points even when the disclosure is negative.

Sample size	The challenge study consisted of three groups of rhesus macaques with 6 animals per group. Based on our experience with SIV in rhesus macaques, this sample size provides power to determine differences in protective efficacy of antibody administered group compared with the control group.
Data exclusions	One animal was excluded in the survival analysis. This animal was mistakenly detected as having detectable viral load after fourth challenge and was therefore not subjected to additional challenges. However, its viral load was undetectable since then, and the viral load repeat for the infection timepoint was negative.
Replication	Virologic and serological measures were performed in duplicate. Technical replicates were minimally different.
Randomization	The animals were balanced for age and gender and otherwise randomized into allocation groups.
Blinding	The viral load measurement and serological measure were performed blinded.

Reporting for specific materials, systems and methods

We require information from authors about some types of materials, experimental systems and methods used in many studies. Here, indicate whether each material, system or method listed is relevant to your study. If you are not sure if a list item applies to your research, read the appropriate section before selecting a response.

Materials & experimental systems

n/a	Involved in the study	
[ ]	[x]	Antibodies
[ ]	[x]	Eukaryotic cell lines
[x]	[ ]	Palaeontology and archaeology
[ ]	[x]	Animals and other organisms
[x]	[ ]	Human research participants
[x]	[ ]	Clinical data
[x]	[ ]	Dual use research of concern

Methods

n/a	Involved in the study	
[x]	[ ]	ChIP-seq
[ ]	[x]	Flow cytometry
[x]	[ ]	MRI-based neuroimaging

Antibodies

Antibodies used	APC-Cy7 mouse anti-human CD3 (clone SP34-2; BD Biosciences; Cat#557757); APC-Cy7 mouse anti-human CD4 (clone OKT4; Biolegend; Cat#317418); APC-Cy7 mouse anti-human CD8 (clone RPA-T8; BD Biosciences; Cat#557760); APC-Cy7 mouse anti-human CD14 (clone M5E2; BD Biosciences; Cat#561384); PerCP/Cy5.5 mouse anti-human CD20 (clone 2H7; Biolegend; Cat#302326); BV421 mouse anti-human IgG (clone G18-145; BD Biosciences; Cat#562581); PE mouse anti-human IgM (clone MHM-88; Biolegend; Cat#314508); Goat anti monkey IgG (H+L) (BioRad; Cat#AAI42); Alkaline phosphatase-conjugated goat anti-human IgG Fcγ antibody (Jackson ImmunoResearch; Cat#109-055-190); 6x His tag monoclonal antibody (Invitrogen; Cat#MA1-21315); CD4-IgG2 (Progenics; Cat#PRO542); Mouse anti-rhodopsin (C9) monoclonal antibody (EMD Millipore, Cat#6B9575); 6x His tag monoclonal antibody (Invitrogen, Cat#MA1-21315); Goat anti-human IgG-HRP antibody (SouthernBiotech, Cat#2045-05)
Validation	All mAbs used according to manufacturer's instructions and/or previously published methods.

Eukaryotic cell lines

Policy information about cell lines

Cell line source(s)	TZM-bl cells (NIH AIDS Reagent Program); HEK293T cells (ATCC); FreeStyle HEK293 cells (ThermoFisher); Expi293F cells (ThermoFisher); Irradiated 3T3msCD40L cells (NIH); HEK293S cells (ATCC)
Authentication	Commercially purchased

Mycoplasma contamination	All cell lines were tested negative for mycoplasma contamination.
Commonly misidentified lines (See ICLAC register)	No commonly misidentified cell lines were used in the study.

Animals and other organisms

Policy information about studies involving animals; ARRIVE guidelines recommended for reporting animal research

Laboratory animals	18 Indian-origin rhesus macaques (Macaca mulatta) between 2- to 6-year-old were used (11 males and 7 females) in the antibody-mediated protection study.
Wild animals	No wild animals were used in the study.
Field-collected samples	No field collected samples were used in the study.
Ethics oversight	All procedures were previously approved by the University of Wisconsin Graduate School Animal Care and Use Committee (animal welfare assurance number 16-00239 [A3368-01]; protocol number G005248).

Note that full information on the approval of the study protocol must also be provided in the manuscript.

Flow Cytometry

Plots

Confirm that:

- [x] The axis labels state the marker and fluorochrome used (e.g. CD4-FITC).
- [x] The axis scales are clearly visible. Include numbers along axes only for bottom left plot of group (a 'group' is an analysis of identical markers).
- [x] All plots are contour plots with outliers or pseudocolor plots.
- [x] A numerical value for number of cells or percentage (with statistics) is provided.

Methodology

Sample preparation	Cryopreserved rhesus peripheral blood mononuclear cells were thawed, washed, and stained for FACS sorting
Instrument	BD FACSMelody or BD FACSFusion was used for cell sorting
Software	Flow cytometric data was analyzed using FlowJo (v10.7.1), index-sort was analyzed using Prism 9.0
Cell population abundance	A total of 8470 antigen-specific memory B cells were single-cell sorted and expanded in vitro
Gating strategy	After gating for lymphocyte (FSC-A/SSC-A) singlets (FSC-H/FSC-W, SSC-H/SSC-W), CD3-CD4-CD8-CD14-CD20+IgM-IgG+ antigen-specific memory B cells were single-cell sorted

- [x] Tick this box to confirm that a figure exemplifying the gating strategy is provided in the Supplementary Information.
